# Micro(nano)plastic and Related Chemicals: Emerging Contaminants in Environment, Food and Health Impacts

**DOI:** 10.3390/toxics12100762

**Published:** 2024-10-20

**Authors:** Juliana G. R. de Carvalho, Helga Coelho Augusto, Ricardo Ferraz, Cristina Delerue-Matos, Virgínia Cruz Fernandes

**Affiliations:** 1 Ciências Químicas e das Biomoléculas, Escola Superior de Saúde, Instituto Politécnico do Porto, Portugal, Rua Dr. António Bernardino de Almeida 400, 4200-072 Porto, Portugal; 10210347@ess.ipp.pt (J.G.R.d.C.); rferraz@ess.ipp.pt (R.F.); 2 Cofisa—Conservas de Peixa da Figueira, S.A., Terrapleno do Porto de Pesca—Gala, 3090-735 Figueira da Foz, Portugal; helgacaugusto@cofisa.pt; 3 LAQV-REQUIMTE, Departamento de Química e Bioquímica, Faculdade de Ciências da Universidade do Porto, 4169-007 Porto, Portugal; 4 Centro de Investigação em Saúde Translacional e Biotecnologia Médica (TBIO)/Rede de Investigação em Saúde (RISE-Health), Escola Superior de Saúde, Instituto Politécnico do Porto, Rua Dr. António Bernardino de Almeida 400, 4200-072 Porto, Portugal; 5 REQUIMTE/LAQV, Instituto Superior de Engenharia do Porto, Instituto Politécnico do Porto, Rua Dr. António Bernardino de Almeida 431, 4249-015 Porto, Portugal; cmm@isep.ipp.pt

**Keywords:** plastic waste, polymers, additives, biological effects

## Abstract

Microplastic pollution is a problem of increasing concern in food, and while food safety issues around the world are serious, an increasing number of food safety issues related to microplastics have become the focus of people’s attention. The presence of microplastics in food is a worldwide problem, and they are present in all kinds of foods, foods of both animal and plant origin, food additives, drinks, plastic food packaging, and agricultural practices. This can cause problems for both humans and the environment. Microplastics have already been detected in human blood, heart, placenta, and breastmilk, but their effects in humans are not well understood. Studies with mammals and human cells or organoids have given perspective about the potential impact of micro(nano)plastics on human health, which affect the lungs, kidneys, heart, neurological system, and DNA. Additionally, as plastics often contain additives or other substances, the potentially harmful effects of exposure to these substances must also be carefully studied before any conclusions can be drawn. The study of microplastics is very complex as there are many factors to account for, such as differences in particle sizes, constituents, shapes, additives, contaminants, concentrations, etc. This review summarizes the more recent research on the presence of microplastic and other plastic-related chemical pollutants in food and their potential impacts on human health.

## 1. Introduction

Plastics are frequently used in a variety of applications in our everyday lives, and they constitute a current source of pollution in the environment through microplastics (MPs), which are particles smaller than 5 mm [1]. Fuller et al. (2022) estimated that in 2019, 9 million people died from pollution (approximately 70% of these deaths were due to household and ambient air pollution) [2]. The safety and toxicity of MPs present throughout the environment have drawn the attention of ecotoxicologists to their safety and toxicity. MPs are resistant to degradation and have adsorbent properties, all of which threaten ecosystems, from oceans to soils to insect digestive tracts, etc. [3]. Microplastic contamination of the aquatic and terrestrial environments jeopardizes biodiversity, which has negative consequences in terms of food security, environmental protection, and, consequently, human health [4].

There were 400.3 MMTs of plastics produced globally in 2022, a 2.5% increase over 2021 [5]. Plastic production has grown significantly since the 1950s. The combination of versatility and affordability accounts for the continuous growth of plastic production. In 2020, Europe was responsible for 15% of the total plastics produced worldwide [6]. The plastics polyvinyl chloride (PVC), polystyrene (PS), polypropylene (PP), and polyethylene (PE) account for more than 60% of total European plastic demand [7]. Plastics that have not been properly disposed of can cross-link with other toxic substances and potentially spread to humans via inadvertent dietary consumption [8]. Plastic particles might be harmful for many reasons; we highlight three: (1) because of their complex chemical characteristics; (2) because of the release of persistent organic pollutants that have been adsorbed to the plastics; and (3) because of the leaching of additives from the plastics [9]. Some examples of contaminants adsorbed on plastic packaging released into food are heavy metals and highly toxic phthalates [10]. The common types of plastic waste frequently found in various locations globally are fragments, fibers, and films [11].

The internal uptake and external adsorption of micro(nano)plastics (MNPs) in plants have been reported to be related to plastic shape and size, with internalization limited to nanosized and very small MPs [12]. Many studies have reported the presence and effects of MNPs in plants [13,14,15], animals [16,17], and food [18,19,20,21]. More recently, a study reported harm caused by MPs from COVID-19 face masks [4].

**Figure 1:**
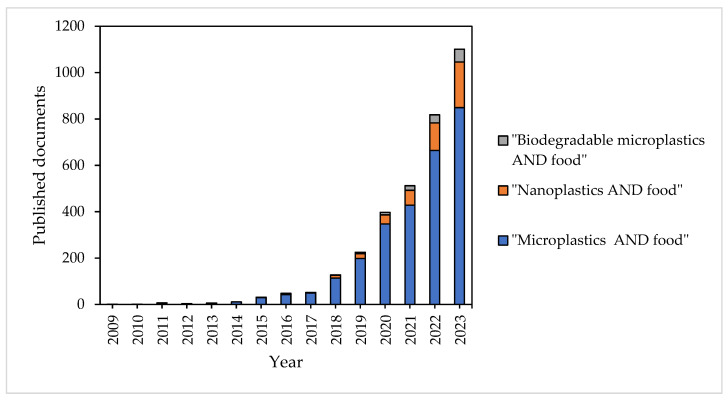
Published documents in the Scopus database.

The first publication related to MPs in food found in the Scopus database was published in 2009 [22]. But in recent years, the number of published documents regarding “microplastics” or “nanoplastics” in food has significantly increased, as shown in Figure 1. However, the number of publications concerning MPs is still not comparable to that of other pollutants of emerging concern, such as heavy metals, pharmaceuticals, plastic additives, and pesticides. Additionally, biodegradable MPs, a variety of MPs, have also been an emerging concern among scientists, and publications relating them to food have almost doubled every year since 2019 (Figure 1).

The scientific community’s lack of agreement on standardized techniques has resulted in a serious deficit of properly comparable data for understanding the environmental distribution, fate, transport, and food levels of MPs and their implications for human health [23]. Therefore, there is an urgent need to comprehend extraction and separation techniques and also to determine a single approach for investigating MPs in food samples [20].

The majority of the literature research confirms that MNPs have adverse impacts on human health [24], although, still, few studies [24] exist. Moreover, models such as mouse and human cells have already been tested, and microplastic toxicity has been confirmed. This review aims to summarize the presence of MPs and nanoplastics in food and their potential effects on human health.

## 2. Microplastics (MPs), Biodegradable Microplastics (BMPs), and Nanoplastics (NPs)

MPs are categorized as primary (beads/pellets used in cosmetics and personal care products), or secondary (fragments of large plastic materials) [1]. The formation of secondary MPs, or tiny plastic fragments, can be caused by a combination of physical abrasion, UV radiation, and microbiological degradation of the environment [25,26].

Plastic waste released into the environment will deteriorate under mechanical, physicochemical, and biological stresses into MPs (<5 mm) or even nanoplastics (NPs, < 100 nm), which will interact with organisms [27]. This plastic can be categorized in terms of sizes such as macro, >25 mm; meso, from 5 to 25 mm; large micro, from 1 to 5 mm; small micro, from 20 µm to 1 mm; and nano, from 1 to 1000 nm [28]. MPs and NPs are referred to together as MNPs [27]. MNPs are also classified according to their form and shape, which include pellets, pieces, fibers, film, rope and filaments, microbeads, sponges or foam, and rubber [29]. Natural sorting mechanisms cause some microplastic forms to predominate in specific situations. Fibrous MPs, for example, are the most common form among fibers, fragments, and granules [30]. The polymers from which the MPs are mostly derived and their characteristics are shown in Table 1.

The main sources of MNPs are consumer-care products, raw industrial materials, fish nets, food packaging [20], and wastewater treatment plants (WWTPs) [3]. Biosolids from WWTPs are a significant source of MPs for agricultural soils treated with biosolids, and wastewater from the laundry of synthetic clothes is a main source of fibers [35]. MNPs are found in different colors, such as transparent, white, orange, red, blue, gray, brown, green, yellow, pink, and crystalline [20]. The most common MPs are fibers and fragments from polyethylene (PE) or polyethylene terephthalate (PET) [36,37,38,39]. PE is one of the most widely used synthetic polymers due to its exceptional thermal, chemical, and processing capabilities, which justifies its abundance in the environment, especially in marine ecosystems [40].

Biopolymers (BPs) are polymers in which enzymes and microorganisms can break them down into CO_2_, H_2_O, CH_4_, and biomass [41]. BPs can be categorized into natural polymers and synthetic polymers based on how they are made. Plastics made from renewable resources, such as plants and biomass, such as polylactic acid (PLA), polyhydroxyalkanoates (PHA), poly-3-hydroxybutyrate (P3HB) [33], starch-based plastics, cellulose-based plastics, protein-based plastics, and others, are primarily classified as natural polymers [42]. Synthetic polymers, such as polybutylene adipate terephthalate (PBAT), polycaprolactone (PCL), and polybutylene succinate (PBS), and others, are produced from nonrenewable resources such as petroleum or gas [43,44]. Some BPs are not biobased, such as PCL made from fossil fuels, and certain bioplastics that are not biobased, such as biopolyethylene and biopolyvinyl chloride. BPs are frequently combined with additional substances and additives [45]. Owing to their high degradability, they are mostly employed in the production of disposable goods, including biodegradable rubbish bags, agricultural mulch, food or food service items, and packaging for perishable commodities such as fresh fruit [43,46]. Additionally, they can be used in medical applications as bioabsorbable polymers, such as in medication capsules, biodegradable screws or plates for mending and repairing ligaments, and wound sutures, and as materials for 3D printers [47].

The biodegradation process of BPs depends on biotic and abiotic factors, such as oxygen, temperature, humidity, and specific microorganisms; however, nature cannot always provide such conditions, and when BPs enter a soil system, MPs may form that are similar to conventional plastics and remain there for a long period of time [42]. Owing to the easier degradation of BPs, more MPs may be created in the same length of time, which would result in even more severe soil microplastic contamination [48,49].

For a better understanding, it is essential to develop biodegradability standards that account for both large-scale breakdown and the formation of MPs and NPs, as well as BMPs. Studying BMP degradation across different real-world environments, including soil, water, and air, is crucial [50]. This is especially important as the use of biodegradable plastics increases in daily life and agriculture, leading to their greater presence in the environment.

### 2.1. Additives in Microplastics

The type of additives used varies across and within plastics: PVC requires the most additives, followed by PE, PP, and PS [51]. Approximately 100 chemicals, including phthalates (plasticizers), have been detected in 120 food-contact plastic goods (e.g., bottles and bags) [52]. Table 2 below shows the functions of the main plastic additives. A total of 10 additives, such as plasticizers (phthalates and adipates), antioxidants (bisphenol B), and phosphorous flame retardants, were found in food containers made of polymers (PPs) and biopolymers (PLAs) [53]. Organophosphite antioxidants (OPAs) and organophosphate esters (OPEs) are found in food contact materials (FCMs) [54]. Phthalic acid ester (PAE) concentrations were found to be correlated with the abundance of ingested MPs in fish species [55].

Plastic additives (PAs) are found in baby food, e.g., baby formulas, cereals, purees, and meat products [56]. Plasticizers, such as diisobutyl phthalate and dibutyl phthalate, were found in samples of spices and roasted chicken meat [57]. Phthalates are also found in frequently consumed food products such as bread, apples, salami, and cheese [58,59]. Phthalates and di-ethylhexyl adipate (DEHA) were found in commercial beer, with an average concentration of 5.8 µg/L [60]. Other phthalates with concentrations as high as 61.56 µg/L have also been detected in regional beers [61]. Phthalates have also been detected in coffee samples obtained from capsules [62,63]. Some of these substances are known as endocrine disruptors because they interfere with the generation, release, transport, metabolism, binding, or removal of natural hormones in the body, which results in endocrine dysfunction in both humans and animals [57].

Antimony trioxide (Sb_2_O_3_) is a common input applied in the manufacture of PET. Its presence in bottled mineral water has been detected [64] at concentrations up to 7.12 ± 0.34 µg/L when it is stored at 60 °C for long periods [65].

**Table 2:** Microplastic additives, their functions, and the common percentages present in the plastic material (adapted from [66]).

Function	Substance Name	Percentage (%)
Light stabilizers	Resorcinol	
	Octabenzone	0.2–5.0
	2-(2H-benzotriazol-2-yl)-4,6-bis(1-methyl-1-phenylethyl)phenol	0.2–5.0
	N-(2-ethoxyphenyl)-N′-(2-ethylphenyl)oxamide	0.7
Nucleating agents	Sodium benzoate	0.2
	Fumes, silica (flame retardant)	n.a.
	2,2′-Methylene bis-(4,6-di-tert-butylphenyl) sodium phosphate	0.2
Antistatic	Sodium acetate	n.a.
	Zinc oxide	5
	Disodium tetraborate, anhydrous	5
	Phosphoric acid, dodecyl ester, potassium salt	n.a
Heat stabilizers	Dibutyltin dilaurate	3
	Triphenyl phosphite	3
	Pentalead tetraoxide sulphate	2
	Diisodecyl phenyl phosphite	3
Antioxidants	6,6′-Di-tert-butyl-4,4′-butylidenedi-m-cresol	0.5
	6,6′-di-tert-butyl-4,4′-thiodi-m-cresol	n.a
	Dioctadecyl 3,3′-thiodipropionate	0.25–3.0
	2,4-Bis(octylthiomethyl)-6-methylphenol	0.015–0.2
Pigments agents	Perylene-3,4:9,10-tetracarboxydiimide	2
	Chromium (III) oxide	1
	Zinc sulphide	2.0–10.0
	Carbon black	2.5–40.0
	2,9-Dichloro-5,12-dihydroquino[2,3-b]acridine-7,14-dione	2
Flame retardants	Triethyl phosphate	10
	Melamine	25
	Cyanuric acid	n.a.
	Diantimony trioxide	8
	Aluminum sodium tetrahydroxide	n.a.
Plasticizers	Tributyl-O-acetyl citrate	10.0–35.0
	Triethyl citrate	10.0–35.0
	2,2′-Ethylenedioxydiethyl bis(2-ethylhexanoate)	n.a.
	Triphenyl phosphate (flame retardant)	2
	Amides, C16-C18 (even), N,N′-ethylenebis	1

### 2.2. Other Contaminants Adhered to Microplastics

In addition to the migration and diffusion of additives from plastic materials to the surface and their release into the environment, the reverse process, i.e., compound adsorption on MNPs, has been observed [67].

Microplastics can act as adsorbents in the environment for a wide range of pollutants, such as heavy metals, and persistent organic pollutants (POPs), like organochlorinated pesticides (OPCs), polycyclic aromatic hydrocarbons (PAHs), polychlorinated biphenyls (PCBs), and dichlorodiphenyltrichloroethane (DDT) [8,27]. PCBs and PAHs have been detected in beach pellets at concentrations of up to 93 and 1592 ng/g, respectively [68,69]. The combustion of land-based biomass was identified as one source of PAHs found in microplastics [70]. The sorption of organic chemicals by MNPs is dependent on pH, salinity, ionic strength, degree of crystallinity, surface weathering, chemical characteristics, and polymer type [71,72]. There are theories suggesting that MPs act as sinks rather than as increasing levels of contaminants in marine life [51]. MPs can also function as carriers of hazardous microbes, such as toxic microalgae [73]. A study carried out with six different MPs assessed the adsorption of α-endosulfan and reported that low-density polyethylene (LDPE) (particle size < 300 µm) could adsorb approximately 0.4 mg/g of α-endosulfan from water [74].

Numerous studies have revealed that MPs may serve as carriers to adsorb various antibiotics such as sulfadiazine, amoxicillin, ciprofloxacin, trimethoprim, and tetracycline, among others [75], through a variety of processes such as electrostatic and hydrophobic interactions [76,77]. Metals and heavy metals have been detected in microplastics worldwide, with Ti, Al, Br, Fe, and Pb being detected at the highest levels [78,79]. The arsenic concentration in MPs reached a value of 6.53 mg/kg, and factors such as organic matter, iron hydroxides, and plastic additives may increase the As adsorption onto MPs [80]. Metal concentrations were found to be higher in samples associated with foam plastic (PS, PUR, PEVA) than in those associated with hard plastic (PE, PP, PET) [81]. Metal desorption is a major problem, and researchers have discovered that the low pH of the digestive system and gut may increase the desorption of toxic metals, causing them to accumulate in the body [82].

To fully understand the behavior and mechanism of MP adsorption of antibiotics, it is necessary to explore how pH, salinity, and other conditions impact the adsorption process [83]. Pathogens and biotoxins have also been identified in MP biofilms, posing a threat to human health [84]

### 2.3. Methods to Extract, Identify, and Quantify Microplastics

The detection and quantification of micro- and nanoplastics (MNPs) pose significant challenges for researchers worldwide. MPs are difficult to work with as they typically represent a small percentage of the sample. Additionally, multiple techniques are necessary to perform a comprehensive analysis of MP polymer identification, mass quantification, and determination of particle distribution, color, shape, and other characteristics. To avoid contamination of the sample, it is necessary to use glass and metal materials, and quality assurance and quality control processes and clean working areas are necessary.

These methods are used in three main aspects: separation, identification, and quantification. Separation methods are particularly challenging due to the heterogeneous nature of samples. Current separation technologies include flotation and filtration, while digestion processes involve enzymatic treatment and chemical digestion. Flotation, based on the principle of density differences between plastic particles and other sample components, is often used to isolate MPs from sediments, food, and biological samples. Filtration techniques are frequently applied for water samples, where filters with specific pore sizes can trap MNPs for further analysis. Membrane bioreactor filtration has been identified as an effective MP separation technology for water and beverages [20]. As each method has advantages and disadvantages, the combination of analytical techniques to identify MPs may be a better solution since it may optimize and enhance the efficiency of the process [20].

Enzymatic treatment, though less common due to higher costs, is employed to digest organic matter in samples, leaving MNPs intact. Chemical digestion, often using potassium hydroxide (KOH) or hydrogen peroxide (H_2_O_2_), is widely used, with KOH being the most commonly used chemical for sample digestion [20,85]. Other digestive solutions, such as inorganic acids (HNO_3_, HCl, and HClO_4_) and enzymes, have been used; however, these methods have limited application in high-density organic materials and are more expensive [18].

After separation, the MNPs are identified and quantified via several analytical techniques. The identification techniques can include optical detection, scanning electron microscopy (SEM), thermoanalytical methods, Fourier transform infrared spectroscopy (FTIR), Raman spectroscopy, and hyperspectral imaging [20,86]. The most frequent identification method for MPs was found to be FTIR, followed by visual identification and Raman spectroscopy [85,87]. Quantification of MNPs typically involves assessing particle concentration, size distribution, and mass. Techniques such as thermogravimetric analysis (TGA) and pyrolysis/GC/MS can be used for polymer mass quantification, offering insights into the total amount of plastics present in a sample.

Some of these methodologies are detailed in Table 3, which provides an overview of the presence of MPs in food and beverages reported in the literature over the last three years. The table highlights the sizes, shapes, colors, and abundance of MPs, as well as the analytical methods applied, demonstrating the diversity of approaches used for detection and analysis.

At present, there is no globally accepted standardized method for detecting, identifying, and quantifying MNPs in food or other complex matrices [88]. The standardization of sampling methodologies, particle characterization techniques, and analytical methods is urgently needed to improve the comparability and reliability of results [89]. Since standardization is not available, all steps (sampling, treatment, solution preparation, equipment required) must be detailed in future works to allow future developments and improvement of the procedures and to achieve more precise results. This transparency will help the future development of improved protocols and enhance the precision and reproducibility of microplastic research [90].

### 2.4. Presence and Toxicity of Biodegradable MPs and Micro- and Nanoplastics in the Environment

MP pollution is a growing concern not only in marine environments but also in terrestrial settings, where the yearly discharge of plastic waste is believed to be 4–23 times greater than in marine environments. Soil, in particular, is a vast reservoir where MPs accumulate, posing a potential risk to both ecological and agricultural systems [91,92]. Despite this, research on soil MPs is still in its infancy compared to marine environments, with only 7.01% of studies focused on soil MPs by 2020, compared to 47.02% focused on marine MPs [93]. This highlights the need for greater attention to the impacts of MPs on terrestrial ecosystems.

Currently, the primary methods for managing plastic waste are burning, landfilling, and recycling, but each method has drawbacks and is ineffective in reducing MP pollution [43].

The toxicological implications of MNPs in freshwater and marine organisms include bioaccumulation; histopathological impacts; survival, growth, and development; oxidative stress; genotoxicity; and reproductive toxicity [27].

The noxious effects of MNPs on organisms are mostly determined by particle size [94], type, charge, and concentration; environmental and biological aspects; and the related toxicity of the adsorbed pollutants [27].

MPs with a diameter of <20 µm have been shown to permeate organs, whereas MPs with a diameter of <10 µm have been shown to penetrate cell membranes and breach the placental barrier in exposed cells or laboratory animals. Despite this, little is known about the harmful consequences of MPs in humans, which may vary on the basis of characteristics like MP type, size, shape, concentration, and charge, among others [95].

MPs have been reported to inhibit the survival, fecundity, and population fitness of the waterflea *Daphnia* [96]. Exposure to MPs decreases the gut digestive enzyme activities of fish and has caused disorders of hepatic lipid metabolism [97,98]. MPs may induce an abnormal and lethargic behavior, promote reactive oxygen species (ROS) production, induce anemia, and affect the immune system of fish; however, as most studies have been conducted under extremely high exposure scenarios, more research on the toxic effects of MPs under realistic exposure scenarios is needed [99]. MPs have been shown to affect a variety of plant species, including lettuce, wheat, broad beans, and maize, by influencing seed germination, root development, and overall plant growth [42]. In recent years, biodegradable MPs (BMPs) have been touted as a more environmentally friendly alternative to conventional MPs. However, emerging research suggests that BMPs may pose an equal or even greater risk to ecosystems under certain conditions. BMPs can break down into smaller, potentially more harmful particles, and their degradation products can interact with environmental pollutants, enhancing their toxicity. Moreover, BMPs may also be more likely to bioaccumulate in organisms due to their tendency to break down in the presence of moisture or microbial activity. Consequently, BMPs could have higher potential for bioavailability in soils, posing risks to soil-dwelling organisms and plants [100].

### 2.5. Occurrence of MPs in Food

There are preliminary findings on the existence of MPs in seafood, poultry, terrestrial snails, a small number of fruits and vegetables, salt, honey, sugar, and water; and a select group of alcoholic drinks, such as beer and wine. Since there is no verified technique available, the validity of these data is, nonetheless, in doubt [101]. The presence of MPs has been detected in different categories of foods, such as beverages, condiments, honey, meat, seafood, and vegetables, with concentrations varying substantially by orders of magnitude [85]. Worldwide findings concerning the presence of MPs in food are discussed in the literature [19,102]. Infant exposure has been a major problem because of contamination from the diet, feeding bottles, and other elements. Microplastics have been detected in canned and box infant milk powders, with the latter being the second most common form of microplastics present [103]. The presence of MNPs in food can be explained not only by aquatic contamination and its trophic transfer in the food chain, but also by the food packing and other plastic-contact sources. A plastic teabag releases approximately 11.6 billion MPs and 3.1 billion NPs into a single drink cup [104]. Another study reported that take-out food containers have from 3 to 29 MPs/container and estimated that people who order take-out meals 4–7 times per week may ingest 12–203 pieces of MPs on the basis of the prevalence of MPs in take-out box [105]. An estimation of 18,500 microplastic particles ingested per year was also reported, considering the weekly consumption of takeaway meals [106].

Table 3 shows the presence of MPs in food and beverages reported in the literature in the last 3 years, by size, shape, color, and abundance, including the method of analysis applied.

**Table 3:** Recent publications on the presence of MNPs in food.

Sample	Source	Digestion	Qualitative/Quantitative Analysis	Qualitative Info	Quantity Info	Ref.
Bivalves: *Ostrea. Denselamellosa* *Sinonovacula. Constricta*	Xiangshan Bay, China	10% KOH + 30% H_2_O_2_, 24 h at 60 °C	Optical/Microscope + μ-FTIR	Fiber.	0.31 ± 0.10 0.21 ± 0.05 0.36 ± 0.07 (items/g)	[107]
Shrimp	Xiangshan Bay, China	10% KOH + 30% H_2_O_2_, 24 h at 60 °C	Optical/Microscope + μ-FTIR	Fiber.	0.25 ± 0.08 items/g	[107]
Fish: *Konosirus punctatus* *Larimichthys crocea*	Xiangshan Bay, China	10% KOH + 30% H_2_O_2_, 24 h at 60 °C	Optical/Microscope + μ-FTIR	Fiber.	0.044 ± 0.025 0.008 ± 0.006 items/g	[107]
Fish	Bangladesh	10% KOH, 72 h at 40 °C	Optical/Microscope	Mostly fiber (50%), fragment (15%), and line (12%). Mostly 300 to 1500 μm. Most colors were transparent (30%), gray (26%) and black (23%).	7 to 51 particles/fish	[108]
Fish	Iran	10% KOH, 48 h at 60 °C	Optical/Microscope + Staining and Fluorescence Microscope + SEM-EDX	Mainly fibers followed by fragments and synthetic microbeads. Mostly <500 μm in light colors.	11.4 MP items per fish	[109]
Fish	Pakistan	10% H_2_O_2_ overnight at 60 °C	Optical/Microscope	Microfibers and microfragments.	~6.62 items/individual	[110]
Salted and dried fish	West coast of India	Mostly Nitric acid (69 %) or sodium hydroxide (10 %) or hydrogen peroxide (30 %) 72 h at 60 °C	Optical/Microscope + Staining + μ-FT-IR	Mostly <100 μm 47.21 %) and by 100–250 μm size group (23.98 %). Mostly fragments and fibers. Mostly translucent and black.	35.57 ± 10.4 to 61.20 ± 21.8 items/g of dried fish	[111]
Seafood varieties	Sri Lanka	30% H_2_O_2_ 24–48 h at 65 °C	Optical/Microscope + Staining + m-FT-IR	Mostly LDPE, PP, HDPE, Nylon-66, and PS. Mostly fibers (52%) and fragments (19%). Mostly blue (69%) and black (17%).	0.04 ± 0.02 MP/g to 1.8 ± 0.21 MPs/g	[112]
Eggs		10% H_2_O_2_ 12/24/48 h at 60 °C	Optical/Microscope + Fluorescence + ATR-FT-IR + FESEM-EDX	Spherical and 50–100 μm.	11.67 ± 3.98 particles/egg	[113]
Seaweed	Korea	35% H_2_O_2_ 72–120 h	Optical/Microscope + FT-IR	Mostly PP and PE, mostly 20–99 μm.	0.20 to 14.30 particles/g	[114]
Honey	Korea	Ethanol and H_2_O_2_	Optical/Microscope + FT-IR	Mostly PP and PE, mostly 20–99 μm.	n. d. to 46.0 particles/L	[114]
Infant milk powder: boxed and canned	China, the Netherlands, Ireland, China, Switzerland, France, and New Zealand	Artificial gastric juice for 3 h at 37 °C	FT-IR	Mostly fragment and fibers. Mostly PE and PET. Average of 139 ± 343 μm and 193 ± 415 μm for boxed and canned, respectively.	1 ± 1 to 11 ± 1 items/100 g	[103]
Soft drinks: PET and Tetra Pak bottles	Turkey	-	Optical/Microscope + FT–IR	Mostly PA and PET. Mostly 50–100 μm. Mostly fiber (60%) and fragment (34%). Mostly transparent (57%) and blue (28%).	5 to 18 polymers/sample	[115]
Beer	Korea	-	Optical/Microscope + FT-IT	Mostly PP and PE, mostly 20–99 μm.	0.01 to 1.02 particles/g	[114]
Food ice cubes	Mexico City	30% H_2_O_2_ 1 h at 65 °C	Epifluorescence Optical/Microscope + SEM-EDX + ATR-FTIR	Fibers (87%), fragments (12.7%), and films (0.3%). Mostly PP and PE.	19 ± 4 to 178 ± 78 items/L	[116]
Mineral water in PET bottles	Iran	-	Optical/Microscope + ATR-FTIR + Raman Microscopy	Mostly fragment (93%). Mostly PET, PS, and PE, 1280–4.200 μm. Mostly transparent, black.	0 to 36 particles/L	[117]
Mineral water in PET bottles	China	-	Optical/Microscope + μ-FTIR + SEM	Mostly fiber and fragment. Mostly PET, PE, PS and PA. Mostly 0.050–0.300 mm.	2 to 23 particles/bottle	[118]

Visual observation via either microscopy or SEM is the most common method applied for MP detection in food, followed by FTIR. The most common types of MPs in fibers and fragment shapes were PE, PP, and PET. The quantities ranged from 0.008 to 61.20 items/g and from 0 to 178 particles/L. The more recent studies are mostly concentrated in Asia, more specifically in China, India, and Korea. The digestion of food and beverage samples is usually carried out with either 10% KOH or 30% H_2_O_2_, or both.

Three mechanisms have been identified as contributors to the release of MPs from plastic bottled in water: the filling process, opening/closing, and squeezing [117]. Adults in China are expected to drink 0.274 MPs/kg daily, which can vary according to consumption behavior [118] but can also be extended to other countries that are high consumers of bottled water. Researchers have estimated that, globally, human ingestion of 0.1 to 5 g of MPs per week occurs through different exposure pathways [96].

### 2.6. Trophic Transfer in the Food Chain

Small plastic particles have been found in the digestive tracts of creatures from various trophic levels, impacting more than 690 marine species [89]. The authors affirm that the physical and chemical characteristics of MPs make it easier for pollutants to stick to the surface of the particles, acting as a vehicle for toxins to reach organisms after ingestion; however, the bioaccumulation parameters for higher-trophic-level animals, and the effects on larger marine food webs, are still unknown.

The transfer of contaminants to marine organisms can take place either directly, with MPs carrying POPs adhering to the organism’s external (such as its skin or skeleton) or internal surfaces (such as its gut or gill walls), or indirectly, with MPs absorbing the contaminants from aqueous phases such as water (external exposure) or organismal fluids (internal exposure) [51]. Trophic transfer is an important pathway for MP exposure that might have negative impacts on sensitive developmental stages [119].

Three routes allow MNPs to enter an animal’s body: (1) through the digestive tract through food and drink, (2) through the lungs, and (3) through the skin surface [17].

**Figure 2:**
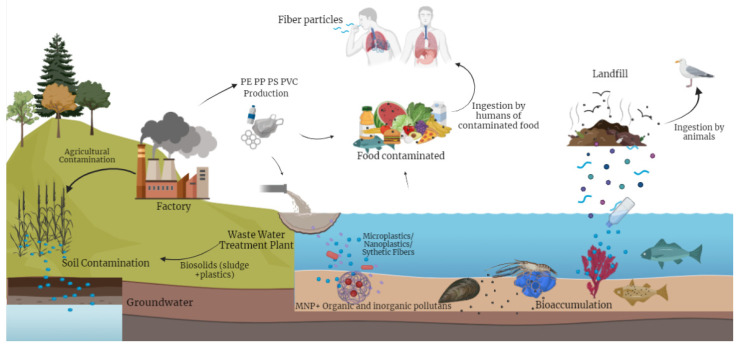
Scheme of the sources and interactions of MNPs and additives in the environment. Created by BioRender.com.

The presence of the NPs in the digestive systems of higher-trophic-level species and their adhesion to the surface of primary producers were both verified by microscopic examination [120]. The results revealed that fish exposed directly to NPs presented histopathological abnormalities in their livers and that the NPs also entered the embryonic walls and were found in the yolk sacs of juveniles after hatching, which demonstrated that NPs are quickly transmitted through the food chain. The effects of plastic and MNPs in animals have been widely investigated. More recently, Charlton-Howard et al. (2023) referred to plasticosis, a new fibrotic disease induced by plastic [121]. Figure 2 illustrates the sources and interactions of MNPs in the environment.

## 3. Presence in Humans and Health Impacts

Human body monitoring research from the late 1990s reported the presence of plastic fibers in lung tissue, which indicated that MPs were deposited or accumulated in the lungs [122]. Approximately 20 years later, investigations have detected the presence of MPs in the human placenta [123] and breastmilk [124], and the first measurement of plastic in human blood was quantified as 1.6 µg/mL [125]. The first evidence of microplastics in the human testis and semen revealed PS, PE, and PVC in sizes of 20 to 100 µm [126]. MPs were also detected for the first time in human vascular tissues, specifically saphenous vein tissue samples [127], and even in the heart [128].

It is estimated that a person who consumes rice, noodles, and packaged meats regularly consumes approximately 400 g of MPs per year, which translates to approximately 50 plastic bags [85,96].

The effects of MPs in humans are being explored in recent studies. The associated risks to humans exposed to MPs are oxidative stress, translocation, inflammation, accumulation, and toxicity from the additive chemicals [129].

Toxicological knowledge of the potential effects of MNP exposure on human health is still in its early stages [95,129]. Most studies are conducted in animals such as mice, as shown in Table 4.

The expected impacts on human health are wide, considering the reported studies. MPs can cause problems in organs, such as the lungs, kidneys, and heart, or even in more complex components such as DNA and the neurological system.

The most common effects are related to cell toxicity, inhibition in cell production, cell apoptosis, metabolic disorders, and organ malfunction. Microplastic exposure has also been shown to be responsible for modifying animal behavior, such as an increase in anxiety rates.

From the literature, we find that the effects of MPs on health are dependent on size, exposure time, and concentration [145,151,153,154] and that the MNP impacts on the intestinal barrier need more attention [155]. Promising studies are being conducted in human cells and organoids, but still, no papers on human beings have been published.

## 4. Legislation and Initiatives

The growing awareness of microplastic pollution has led to various global initiatives and legislative measures aimed at mitigating its impact. Initiatives such as the creation of the Agenda 2030 by the United Nations (UN) are highly relevant to incentive actions toward the microplastic issue [156]. Among the 17 Sustainable Development Goals (SDGs), several are related, such as 3—Good health and well-being, 6—Clean water and sanitation, 12—Sustainable consumption and production, and 14—Life below water. In 2017, the UN Environment Programme (UNEP) launched the Clean Seas Campaign, which was devoted to ending marine litter and plastic pollution [157]. The United States and the European Commission formally joined the Clean Seas Campaign in 2022, showing their commitment to reducing plastic waste in the oceans.

Some countries have started to create legislation to limit the application of MPs in industry; however, most of them are related to cosmetic and personal care products, which are a source of primary MPs [158].

Much effort has been given to reducing the consumption of plastic bags, which are also sources of MPs, either by banning them or charging for their usage. The pioneers were California State and the United Kingdom, which restricted the usage of plastic bags in 2014 and 2015, respectively. A total nationwide ban has been reported in some countries of Africa (e.g., Cameroon, Morocco, and Kenya), Asia (e.g., China, Taiwan, and Malaysia), and Europe (e.g., France and Italy) [159]. These bans, particularly in developing countries like Kenya, are significant steps toward reducing plastic pollution, though enforcement and compliance vary widely across regions.

Since 2018, the European Commission has adopted a plastic strategy to protect the environment, reduce marine litter, and transform the way plastic products are designed for use and recycled in the European Union [160]. On average, Europe consumes 180 kg of packaging waste per year, which is mostly made of plastic and paper. In 2019, a directive on single-use plastics entered into force in the EU; however, by the beginning of 2022, 60% of the Member States had not yet implemented it [161]. At the end of 2022, the Commission began working on a policy framework for biobased, biodegradable, and compostable plastics.

More recently, the European Commission published the Commission Delegated Decision Directive 2024/1441, supplementing Directive EU 2020/2184, which establishes a methodology for measuring microplastics in drinking water [162]. This is a first step toward the definition of an official protocol to detect and measure microplastics in water samples, which can also serve as a model for food and beverage samples.

In summary, while there are notable efforts worldwide to reduce plastic pollution, the effectiveness and implementation of these policies vary significantly between regions. Countries like those in the EU are leading the way with ambitious regulatory frameworks, but the lack of control, delays in implementation, and inconsistent enforcement reduce their overall impact. In contrast, developing nations face the dual challenges of enforcement and infrastructure, while major economies such as the United States and China still lack comprehensive microplastic-specific legislation. A more coordinated global approach, along with the development of standardized detection methodologies, is urgently needed to tackle the growing problem of microplastic pollution effectively.

## 5. Conclusions and Future Perspectives

The presence of the MPs in the environment and our food is unquestionable. Researchers worldwide have found MPs in different kinds of foods and beverages. Plastic material has been found in different parts of humans, such as lung tissue, placenta, breastmilk, blood, heart, testis, semen, and human vascular tissues, specifically saphenous vein tissue samples. Microplastic pollution is currently underrated, considering the preliminary studies of health effects in animals and potential impacts on human health, such as cell toxicity, inhibition of cell production, cell apoptosis, metabolic disorders, and organ malfunction. Yet, there remains a significant gap in our understanding of how MPs affect human health, particularly over the long term and with chronic exposure. There is already a pathology in seabirds induced by plastics, called *plasticosis* [121], which underscores the need for urgent research into similar impacts on humans.

Rethinking our consumption habits and changing legislation is a baby step toward solving the significant problem we are facing. However, current efforts—such as limiting plastic use and promoting recycling—are still inadequate, given the magnitude of the problem. Even eco-friendly alternatives like bioplastics and existing recycling methods contribute to MP pollution. This highlights the need for innovation in plastic-degrading technologies and mitigation strategies, such as plastic-degrading enzymes [3], advanced oxidation processes, photocatalysis, and nanotechnology-based solutions [163].

Moving forward, several priority areas for investigation emerge. First, there is an urgent need for standardized methods for identifying and quantifying MNPs, including improved sample preparation techniques. Standardization will allow for better comparability of results across studies and regions. Second, more research is needed to understand the long-term effects of MPs on human health, particularly regarding chronic exposure from food and beverages. Studies should focus on the impacts of different concentrations, particle sizes, and polymer types, as well as the interaction between MPs and environmental contaminants or additives. Moreover, the cumulative effects of long-term exposure to MPs in humans, especially in vulnerable populations like children, pregnant women, and individuals with preexisting conditions, should be a research priority. Epidemiological studies investigating the correlation between MP exposure and health outcomes in humans are critically lacking. Without these data, assessing the true scale of the risk remains difficult.

As researchers worldwide report food contamination from MPs in almost all types of foods and beverages, a new question arises: is it possible to make food free from MP contamination? If the answer is yes, then how? This critical question must be addressed through interdisciplinary collaboration, involving food scientists, environmental researchers, policymakers, and the agricultural sector, to develop practical solutions for ensuring safer food production and consumption [164].

## Figures and Tables

**Table 1:** Polymer characteristics of MPs present in the environment.

Microplastic Polymer		Density (g/cm^3^)	Molecular Weight (g/mol)	Applications	Ref.
Polyethylene (PE)		0.90–0.99	30,000–50,000	Plastic bags, straws	[20]
Polypropylene (PP)		0.85–0.95	~67,000	Bottle caps, netting	[20]
Polystyrene (PS)		0.95–1.1	1,00,000–4,00,000	Food containers, foam cups	[20]
Polyamide (PA)		1.02–1.15	224.3	Trap netting	[20]
Polyester (PES)		1.38	4000	Clothes, fibers	[20]
Polyvinyl chloride (PVC)		1.1–1.58	~99,000	Plastic films, cups	[20]
Polyethylene terephthalate (PET)		1.38–1.45	222.24	Bottles	[20]
Polylactic acid (PLA)	Biopolymers	1.24	120,000	Biomedicine	[31,32]
Poly-3-hydroxybutyrate (P3HB)		1.248	206,000	Biotechnology, biomedicine	[33]
Polyhydroxybutyrate (PHB)		1.20	600,000	Veterinary, flasks, pens	[34]

**Table 4:** Health impacts of micro(nano) plastics and related substances.

Model	Material Evaluated	Concentration	Size	Exposure	Health Impact	Source
Mice	MPs and di (2-ethyl) hexyl phthalate (DEHP)	0.1 g/L (MP/MP + DEHP); 200 µm/Kg DEHP	1–10 µm	1 week	Delayed skin healing.	[130]
Mice	Polystyrene MPs	10 mg/L	1–10 µm and 50–100 µm	30 days	Delayed skeletal muscle regeneration.	[131]
Rats	Polystyrene MPs	0.5 mg/L; 5 mg/L and 50 mg/L	0.5 µm	90 days	Damage on the muscle cardiac structure, apoptosis of myocardium and cardiac fibrosis.	[132]
Rats	Polystyrene MPs	0.1%	0.10 µm	14 days	Alterations observed on endpoints in physiological, serum biochemical, hematological, and respiratory function markers.	[133]
Mice	Polystyrene MPs	100 and 1000 µg/L	0.5 and 50 µm	5 weeks	Decrease of the secretion of mucin in gut, induced gut microbiota dysbiosis, induced hepatic lipid metabolism disorder.	[134]
Mice	Polystyrene MPs	0.1 mg/day	5 µm and 20 µm	28 days	Disturbance of energy and lipid metabolism, oxidative stress, alteration of blood biomarkers of neurotoxicity.	[135]
Mice	Polystyrene MPs	100 and 1000 µg/L	5 µm	6 weeks	Intestinal barrier dysfunction, gut microbiota dysbiosis, bile acid metabolism disorder.	[136]
Mice	Polystyrene MPs	0.1 mg/day	5 µm	30 days	Inflammation, apoptosis and oxidative stress, hepatic injury.	[137]
Mice	Tributyltin + microplastics	0.1 mg/day	5 µm	33 days	Inflammation and apoptosis in epidermis.	[138]
Mice	Di (2-ethylhexyl) phthalate (DEHP)	40 µ/Kg	-	17.5 DPC (days post coitum)	Obstruction of follicle assembly progress and interference with their developmental status, increase in DNA damage, and apoptosis in germ cells and/or somatic cells.	[139]
Neonatal rats	Di (2-ethylhexyl) phthalate (DEHP)	60, 300, or 600 mg/day	-	21 days	Reductions in testis weight, germ cell and Sertoli cell toxicity, lung granulomas, inhibition of lung alveolar development	[140]
Mice	Polystyrene MPs	100–1000 µg/L	1 µm	8 weeks	Impaired glucose tolerance and hepatic lipid deposition; alteration in hepatic lipid species.	[141]
Mice	Polystyrene MPs	100 µg/L and 1000 µg/L	0.5 µm, 4 µm, and 10 µm	180 days	Alterations in testicular morphology and reductions in testosterone, LH, and FSH contents in serum, decline in sperm viability and increase in rate of sperm abnormality.	[142]
Mice	Polystyrene MPs	100 µg/L, 1000 µg/L, and 10 mg/L	5 µm	35 days	Sperm quality decline, abnormal testicular spermatogenesis.	[143]
Mice	Polystyrene NP-MPs	100 mg/mL	NPs: 50 nm; MPs: 300 nm, 600 nm and 4 µm	4 weeks	Kidney inflammation, histological damage of kidney, mice weight loss, increase in death rate.	[144]
Pregnant mice	Polystyrene MPs	100 µg/L and 1000 µg/L	0.5 µm and 5 µm	Gestation period	Potential risk of fatty acid metabolism disorder in offspring.	[145]
Pregnant and postnatal mice	Polystyrene MPs	0.5 mg/L, 5 mg/L, and 50 mg/L	0.5 µm	35 and 70 PND (post-natal days)	Testis development disorder and male subfertility, likely regulated by the Hippo signaling pathway and involving an immune reaction.	[146]
Mice	Polyethylene and polystyrene MPs and organophosphorus flame retardants (OPFRs)	10 µg/L and 100 µg/L	0.5–1.0 µm	90 days	Coexposure to MPs and OPFRs increased oxidative stress, induced greater neurotoxicity, and enhanced disruption of amino acid metabolism and energy metabolism.	[147]
Mice	Polyethylene MPs	500 mg/L	Different sizes and shapes (35.46 µm ± 18.17 µm)	7 days	Impacted animal behavior: higher anxiety index, slower locomotion speed, lack of defensive social aggregation, and reduction in risk assessment behavior.	[148]
Human organoids	Polystyrene MPs	0.25 µg/L, 2.5 µg/L, and 25 µg/L	1 µm	48 h	Hepatotoxicity and disruption of lipid metabolism in human pluripotent stem cell-derived liver organoids.	[149]
Human cells	Polystyrene MPs	1 µg/L, 10 µg/L, 20 µg/L, 50 µg/L, 80 µg/L, and 200 µg/L	0.1 µm and 5 µm	12 h	Induction of higher mitochondrial depolarization in human colon adenocarcinoma Caco-2 cells.	[150]
Human cells	Polystyrene MPs	NP25: 30, 25,20, 15, 10, 5, and 2.5 µg/mL; NP70: 300, 220, 160, 100, 60, 30, and 10 µg/mL	NP: 25 nm and 70 nm	2 h, 4 h, and 8 h	Affected the viability, apoptosis, and cell cycles of A549 human lung epithelial cells.	[151]
Human cells	Polystyrene MPs	10 and 1000 µg/cm^2^	1.72 ± 0.26 µm	24 and 48 h	Pulmonary cytotoxicity, pulmonary barrier impairment, and chronic obstructive pulmonary disease.	[152]

## Data Availability

Data for the results presented in this article will be available upon request.

## References

[1] Wang F., Wang B., Duan L., Zhang Y., Zhou Y., Sui Q., Xu D., Qu H., Yu G. (2020). Occurrence and distribution of microplastics in domestic, industrial, agricultural and aquacultural wastewater sources: A case study in Changzhou, China. Water Res..

[2] Fuller R., Landrigan P.J., Balakrishnan K., Bathan G., Bose-O’Reilly S., Brauer M., Caravanos J., Chiles T., Cohen A., Corra L. (2022). Pollution and health: A progress update. Lancet Planet. Health.

[3] Zurier H.S., Goddard J.M. (2021). Biodegradation of microplastics in food and agriculture. Curr. Opin. Food Sci..

[4] Jimoh J.O., Rahmah S., Mazelan S., Jalilah M., Olasunkanmi J.B., Lim L.-S., Ghaffar M.A., Chang Y.M., Bhubalan K., Liew H.J. (2023). Impact of face mask microplastics pollution on the aquatic environment and aquaculture organisms. Environ. Pollut..

[5] Statista Annual Production of Plastics Worldwide from 1950 to 2022. https://www.statista.com/statistics/282732/global-production-of-plastics-since-1950/.

[6] Plastics Europe (2021). Plastics—The Facts 2021 An Analysis of European Plastics Production, Demand and Waste Data.

[7] Plastics Europe (2020). Plastics—The Facts 2020. An Analysis of European Plastics Production, Demand and Waste Data.

[8] Conti I., Simioni C., Varano G., Brenna C., Costanzi E., Neri L.M. (2021). Legislation to limit the environmental plastic and microplastic pollution and their influence on human exposure. Environ. Pollut..

[9] Bouwmeester H., Hollman P.C.H., Peters R.J.B. (2015). Potential Health Impact of Environmentally Released Micro- and Nanoplastics in the Human Food Production Chain: Experiences from Nanotoxicology. Environ. Sci. Technol..

[10] Cherif Lahimer M., Ayed N., Horriche J., Belgaied S. (2017). Characterization of plastic packaging additives: Food contact, stability and toxicity. Arab. J. Chem..

[11] Wagner M., Lambert S. (2018). Freshwater Microplastics.

[12] Mateos-Cárdenas A., van Pelt F.N.A.M., O’Halloran J., Jansen M.A.K. (2021). Adsorption, uptake and toxicity of micro- and nanoplastics: Effects on terrestrial plants and aquatic macrophytes. Environ. Pollut..

[13] Li J., Yu S., Yu Y., Xu M. (2022). Effects of Microplastics on Higher Plants: A Review. Bull. Environ. Contam. Toxicol..

[14] Ge J., Li H., Liu P., Zhang Z., Ouyang Z., Guo X. (2021). Review of the toxic effect of microplastics on terrestrial and aquatic plants. Sci. Total Environ..

[15] van Weert S., Redondo-Hasselerharm P.E., Diepens N.J., Koelmans A.A. (2019). Effects of nanoplastics and microplastics on the growth of sediment-rooted macrophytes. Sci. Total Environ..

[16] Zantis L.J., Carroll E.L., Nelms S.E., Bosker T. (2021). Marine mammals and microplastics: A systematic review and call for standardisation. Environ. Pollut..

[17] Dong X., Liu X., Hou Q., Wang Z. (2023). From natural environment to animal tissues: A review of microplastics(nanoplastics) translocation and hazards studies. Sci. Total Environ..

[18] Kwon J.H., Kim J.W., Pham T.D., Tarafdar A., Hong S., Chun S.H., Lee S.H., Kang D.Y., Kim J.Y., Kim S.B. (2020). Microplastics in Food: A Review on Analytical Methods and Challenges. Int. J. Environ. Res. Public Health.

[19] Udovicki B., Andjelkovic M., Cirkovic-Velickovic T., Rajkovic A. (2022). Microplastics in food: Scoping review on health effects, occurrence, and human exposure. Int. J. Food Contam..

[20] Sridhar A., Kannan D., Kapoor A., Prabhakar S. (2022). Extraction and detection methods of microplastics in food and marine systems: A critical review. Chemosphere.

[21] Mercogliano R., Avio C.G., Regoli F., Anastasio A., Colavita G., Santonicola S. (2020). Occurrence of Microplastics in Commercial Seafood under the Perspective of the Human Food Chain. A Review. J. Agric. Food Chem..

[22] Fendall L.S., Sewell M.A. (2009). Contributing to marine pollution by washing your face: Microplastics in facial cleansers. Mar. Pollut. Bull..

[23] Koelmans A.A., Mohamed Nor N.H., Hermsen E., Kooi M., Mintenig S.M., De France J. (2019). Microplastics in freshwaters and drinking water: Critical review and assessment of data quality. Water Res..

[24] Xu J.-L., Lin X., Wang J.J., Gowen A.A. (2022). A review of potential human health impacts of micro- and nanoplastics exposure. Sci. Total Environ..

[25] Wagner M., Scherer C., Alvarez-Muñoz D., Brennholt N., Bourrain X., Buchinger S., Fries E., Grosbois C., Klasmeier J., Marti T. (2014). Microplastics in freshwater ecosystems: What we know and what we need to know. Environ. Sci. Eur..

[26] Ivleva N.P., Wiesheu A.C., Niessner R. (2017). Microplastic in Aquatic Ecosystems. Angew. Chem. Int. Ed..

[27] Gao D., Liu X., Junaid M., Liao H., Chen G., Wu Y., Wang J. (2022). Toxicological impacts of micro(nano)plastics in the benthic environment. Sci. Total Environ..

[28] Gigault J., Halle A.T., Baudrimont M., Pascal P.-Y., Gauffre F., Phi T.-L., El Hadri H., Grassl B., Reynaud S. (2018). Current opinion: What is a nanoplastic?. Environ. Pollut..

[29] Frias J., Pagter E., Nash R., O’Connor I., Carretero O., Filgueiras A., Viñas L., Gago J., Antunes J., Bessa F. (2018). Standardised Protocol for Monitoring Microplastics in Sediments.

[30] Ding J.-F., Li J.-X., Sun C.-J., He C.-F., Jiang F.-H., Gao F.-L., Zheng L. (2018). Separation and Identification of Microplastics in Digestive System of Bivalves. Chin. J. Anal. Chem..

[31] Backes E.H., Pires L.d.N., Costa L.C., Passador F.R., Pessan L.A. (2019). Analysis of the Degradation During Melt Processing of PLA/Biosilicate^®^ Composites. J. Compos. Sci..

[32] Kamarudin S.H., Abdullah L.C., Aung M.M., Ratnam C.T. (2020). Thermal and Structural Analysis of Epoxidized Jatropha Oil and Alkaline Treated Kenaf Fiber Reinforced Poly(Lactic Acid) Biocomposites. Polymers.

[33] Iordanskii A.L., Bychkova A.V., Gumargalieva K.Z., Berlin A.A., Grumezescu A.M. (2016). Chapter 6—Magnetoanisotropic biodegradable nanocomposites for controlled drug release. Nanobiomaterials in Drug Delivery.

[34] Omnexus BIOCYCLE® 1000 Technical Datasheet. https://omnexus.specialchem.com/product/t-phb-industrial-biocycle-1000.

[35] Crossman J., Hurley R.R., Futter M., Nizzetto L. (2020). Transfer and transport of microplastics from biosolids to agricultural soils and the wider environment. Sci. Total Environ..

[36] Li J., Qu X., Su L., Zhang W., Yang D., Kolandhasamy P., Li D., Shi H. (2016). Microplastics in mussels along the coastal waters of China. Environ. Pollut..

[37] Li J., Yang D., Li L., Jabeen K., Shi H. (2015). Microplastics in commercial bivalves from China. Environ. Pollut..

[38] Tibbetts J., Krause S., Lynch I., Sambrook Smith G.H. (2018). Abundance, Distribution, and Drivers of Microplastic Contamination in Urban River Environments. Water.

[39] Tiwari M., Rathod T.D., Ajmal P.Y., Bhangare R.C., Sahu S.K. (2019). Distribution and characterization of microplastics in beach sand from three different Indian coastal environments. Mar. Pollut. Bull..

[40] Fernández-González V., Andrade-Garda J.M., López-Mahía P., Muniategui-Lorenzo S. (2021). Impact of weathering on the chemical identification of microplastics from usual packaging polymers in the marine environment. Anal. Chim. Acta.

[41] Luyt A.S., Malik S.S. (2019). 16—Can Biodegradable Plastics Solve Plastic Solid Waste Accumulation?. Plastics to Energy, Al-Salem, S.M., Ed..

[42] Fan P., Yu H., Xi B., Tan W. (2022). A review on the occurrence and influence of biodegradable microplastics in soil ecosystems: Are biodegradable plastics substitute or threat?. Environ. Int..

[43] Shen M., Song B., Zeng G., Zhang Y., Huang W., Wen X., Tang W. (2020). Are biodegradable plastics a promising solution to solve the global plastic pollution?. Environ. Pollut..

[44] Ashter S.A. (2016). Overview of biodegradable polymers. Introduction to Bioplastics Engineering.

[45] Rai P., Mehrotra S., Priya S., Gnansounou E., Sharma S.K. (2021). Recent advances in the sustainable design and applications of biodegradable polymers. Bioresour. Technol..

[46] Qin M., Chen C., Song B., Shen M., Cao W., Yang H., Zeng G., Gong J. (2021). A review of biodegradable plastics to biodegradable microplastics: Another ecological threat to soil environments?. J. Clean. Prod..

[47] Shaikh S., Yaqoob M., Aggarwal P. (2021). An overview of biodegradable packaging in food industry. Curr. Res. Food Sci..

[48] Beltrán-Sanahuja A., Benito-Kaesbach A., Sánchez-García N., Sanz-Lázaro C. (2021). Degradation of conventional and biobased plastics in soil under contrasting environmental conditions. Sci. Total Environ..

[49] Liao J., Chen Q. (2021). Biodegradable plastics in the air and soil environment: Low degradation rate and high microplastics formation. J. Hazard. Mater..

[50] Wang F., Xiang L., Sze-Yin Leung K., Elsner M., Zhang Y., Guo Y., Pan B., Sun H., An T., Ying G. (2024). Emerging contaminants: A One Health perspective. The Innovation.

[51] Rodrigues J.P., Duarte A.C., Santos-Echeandía J., Rocha-Santos T. (2019). Significance of interactions between microplastics and POPs in the marine environment: A critical overview. TrAC Trends Anal. Chem..

[52] Qian S., Ji H., Wu X., Li N., Yang Y., Bu J., Zhang X., Qiao L., Yu H., Xu N. (2018). Detection and quantification analysis of chemical migrants in plastic food contact products. PLoS ONE.

[53] Akoueson F., Chbib C., Brémard A., Monchy S., Paul-Pont I., Doyen P., Dehaut A., Duflos G. (2022). Identification of plastic additives: Py/TD-GC-HRMS method development and application on food containers. J. Anal. Appl. Pyrolysis.

[54] Wang L., Xiao Q., Yuan M., Lu S. (2022). Discovery of 18 Organophosphate Esters and 3 Organophosphite Antioxidants in Food Contact Materials Using Suspect and Nontarget Screening: Implications for Human Exposure. Environ. Sci. Technol..

[55] Sambolino A., Iniguez E., Herrera I., Kaufmann M., Dinis A., Cordeiro N. (2023). Microplastic ingestion and plastic additive detection in pelagic squid and fish: Implications for bioindicators and plastic tracers in open oceanic food webs. Sci. Total Environ..

[56] Tan H., Yang L., Huang D., Chen H., Yang Y., Chen D. (2023). Contamination of Baby Foods by Plastic Additives: A Pilot Screening Study. Environ. Sci. Technol. Lett..

[57] Moreira M.A., André L.C., Cardeal Z.d.L. (2015). Analysis of plasticiser migration to meat roasted in plastic bags by SPME–GC/MS. Food Chem..

[58] Van Holderbeke M., Geerts L., Vanermen G., Servaes K., Sioen I., De Henauw S., Fierens T. (2014). Determination of contamination pathways of phthalates in food products sold on the Belgian market. Environ. Res..

[59] Fasano E., Cirillo T., Esposito F., Lacorte S. (2015). Migration of monomers and plasticizers from packed foods and heated microwave foods using QuEChERS sample preparation and gas chromatography/mass spectrometry. LWT-Food Sci. Technol..

[60] Pereira C., Cunha S.C., Fernandes J.O. (2023). Commercial beers: A source of phthalates and di-ethylhexyl adipate. Food Chem. X.

[61] Carnol L., Schummer C., Moris G. (2017). Quantification of Six Phthalates and One Adipate in Luxembourgish Beer Using HS-SPME-GC/MS. Food Anal. Methods.

[62] Domínguez-Hernández C., Ortega-Zamora C., González-Sálamo J., Hernández-Borges J. (2022). Determination of phthalic acid esters and di(2-ethylhexyl) adipate in coffee obtained from capsules. Food Chem..

[63] De Toni L., Tisato F., Seraglia R., Roverso M., Gandin V., Marzano C., Padrini R., Foresta C. (2017). Phthalates and heavy metals as endocrine disruptors in food: A study on pre-packed coffee products. Toxicol. Rep..

[64] de Oliveira L.L.G., Ferreira G.O., Suquila F.A.C., de Almeida F.G., Bertoldo L.A., Segatelli M.G., Ribeiro E.S., Tarley C.R.T. (2019). Development of new analytical method for preconcentration/speciation of inorganic antimony in bottled mineral water using FIA-HG AAS system and SiO2/Al2O3/SnO2 ternary oxide. Food Chem..

[65] Carneado S., Hernández-Nataren E., López-Sánchez J.F., Sahuquillo A. (2015). Migration of antimony from polyethylene terephthalate used in mineral water bottles. Food Chem..

[66] European Chemicals Agency Mapping Exercise—Plastic Additives Initiative. https://echa.europa.eu/mapping-exercise-plastic-additives-initiative.

[67] Gallo F., Fossi C., Weber R., Santillo D., Sousa J., Ingram I., Nadal A., Romano D. (2018). Marine litter plastics and microplastics and their toxic chemicals components: The need for urgent preventive measures. Environ. Sci. Eur..

[68] Arias A.H., Alvarez G., Pozo K., Pribylova P., Klanova J., Rodríguez Pirani L.S., Picone A.L., Alvarez M., Tombesi N. (2023). Beached microplastics at the Bahia Blanca Estuary (Argentina): Plastic pellets as potential vectors of environmental pollution by POPs. Mar. Pollut. Bull..

[69] Karkanorachaki K., Kiparissis S., Kalogerakis G.C., Yiantzi E., Psillakis E., Kalogerakis N. (2018). Plastic pellets, meso- and microplastics on the coastline of Northern Crete: Distribution and organic pollution. Mar. Pollut. Bull..

[70] Fred-Ahmadu O.H., Tenebe I.T., Ayejuyo O.O., Benson N.U. (2022). Microplastics and associated organic pollutants in beach sediments from the Gulf of Guinea (SE Atlantic) coastal ecosystems. Chemosphere.

[71] Fred-Ahmadu O.H., Bhagwat G., Oluyoye I., Benson N.U., Ayejuyo O.O., Palanisami T. (2020). Interaction of chemical contaminants with microplastics: Principles and perspectives. Sci. Total Environ..

[72] Gateuille D., Naffrechoux E. (2022). Transport of persistent organic pollutants: Another effect of microplastic pollution?. WIREs Water.

[73] Casabianca S., Capellacci S., Giacobbe M.G., Dell’Aversano C., Tartaglione L., Varriale F., Narizzano R., Risso F., Moretto P., Dagnino A. (2019). Plastic-associated harmful microalgal assemblages in marine environment. Environ. Pollut..

[74] Martinho S.D., Fernandes V.C., Figueiredo S.A., Delerue-Matos C. (2022). Study of the Potential Accumulation of the Pesticide Alpha-Endosulfan by Microplastics in Water Systems. Polymers.

[75] Naik R.K., Naik M.M., D’Costa P.M., Shaikh F. (2019). Microplastics in ballast water as an emerging source and vector for harmful chemicals, antibiotics, metals, bacterial pathogens and HAB species: A potential risk to the marine environment and human health. Mar. Pollut. Bull..

[76] Fu L., Li J., Wang G., Luan Y., Dai W. (2021). Adsorption behavior of organic pollutants on microplastics. Ecotoxicol. Environ. Saf..

[77] Mei W., Chen G., Bao J., Song M., Li Y., Luo C. (2020). Interactions between microplastics and organic compounds in aquatic environments: A mini review. Sci. Total Environ..

[78] Li W., Lo H.-S., Wong H.-M., Zhou M., Wong C.-Y., Tam N.F.-Y., Cheung S.-G. (2020). Heavy metals contamination of sedimentary microplastics in Hong Kong. Mar. Pollut. Bull..

[79] Kutralam-Muniasamy G., Pérez-Guevara F., Martínez I.E., Shruti V.C. (2021). Overview of microplastics pollution with heavy metals: Analytical methods, occurrence, transfer risks and call for standardization. J. Hazard. Mater..

[80] Mora A., Dueñas-Moreno J., Mahlknecht J. (2023). Microplastics as a vector of arsenic contamination. Curr. Opin. Environ. Sci. Health.

[81] Fred-Ahmadu O.H., Ayejuyo O.O., Tenebe I.T., Benson N.U. (2022). Occurrence and distribution of micro(meso)plastic-sorbed heavy metals and metalloids in sediments, Gulf of Guinea coast (SE Atlantic). Sci. Total Environ..

[82] Khalid N., Aqeel M., Noman A., Khan S.M., Akhter N. (2021). Interactions and effects of microplastics with heavy metals in aquatic and terrestrial environments. Environ. Pollut..

[83] Wang L., Yang H., Guo M., Wang Z., Zheng X. (2023). Adsorption of antibiotics on different microplastics (MPs): Behavior and mechanism. Sci. Total Environ..

[84] Tavelli R., Callens M., Grootaert C., Abdallah M.F., Rajkovic A. (2022). Foodborne pathogens in the plastisphere: Can microplastics in the food chain threaten microbial food safety?. Trends Food Sci. Technol..

[85] Bai C.-L., Liu L.-Y., Hu Y.-B., Zeng E.Y., Guo Y. (2022). Microplastics: A review of analytical methods, occurrence and characteristics in food, and potential toxicities to biota. Sci. Total Environ..

[86] Ivleva N.P. (2021). Chemical Analysis of Microplastics and Nanoplastics: Challenges, Advanced Methods, and Perspectives. Chem. Rev..

[87] Fischer M., Scholz-Böttcher B.M. (2017). Simultaneous Trace Identification and Quantification of Common Types of Microplastics in Environmental Samples by Pyrolysis-Gas Chromatography–Mass Spectrometry. Environ. Sci. Technol..

[88] Anbumani S., Kakkar P. (2018). Ecotoxicological effects of microplastics on biota: A review. Environ. Sci. Pollut. Res..

[89] Carbery M., O’Connor W., Palanisami T. (2018). Trophic transfer of microplastics and mixed contaminants in the marine food web and implications for human health. Environ. Int..

[90] Martinho S.D., Fernandes V.C., Figueiredo S.A., Delerue-Matos C. (2022). Microplastic Pollution Focused on Sources, Distribution, Contaminant Interactions, Analytical Methods, and Wastewater Removal Strategies: A Review. Int. J. Environ. Res. Public Health.

[91] Horton A.A., Walton A., Spurgeon D.J., Lahive E., Svendsen C. (2017). Microplastics in freshwater and terrestrial environments: Evaluating the current understanding to identify the knowledge gaps and future research priorities. Sci. Total Environ..

[92] Chen Y., Leng Y., Liu X., Wang J. (2020). Microplastic pollution in vegetable farmlands of suburb Wuhan, central China. Environ. Pollut..

[93] Yang L., Zhang Y., Kang S., Wang Z., Wu C. (2021). Microplastics in soil: A review on methods, occurrence, sources, and potential risk. Sci. Total Environ..

[94] Ziajahromi S., Kumar A., Neale P.A., Leusch F.D.L. (2018). Environmentally relevant concentrations of polyethylene microplastics negatively impact the survival, growth and emergence of sediment-dwelling invertebrates. Environ. Pollut..

[95] Kannan K., Vimalkumar K. (2021). A Review of Human Exposure to Microplastics and Insights Into Microplastics as Obesogens. Front. Endocrinol..

[96] Senathirajah K., Attwood S., Bhagwat G., Carbery M., Wilson S., Palanisami T. (2021). Estimation of the mass of microplastics ingested—A pivotal first step towards human health risk assessment. J. Hazard. Mater..

[97] Huang J.-N., Wen B., Zhu J.-G., Zhang Y.-S., Gao J.-Z., Chen Z.-Z. (2020). Exposure to microplastics impairs digestive performance, stimulates immune response and induces microbiota dysbiosis in the gut of juvenile guppy (Poecilia reticulata). Sci. Total Environ..

[98] Zhao Y., Bao Z., Wan Z., Fu Z., Jin Y. (2020). Polystyrene microplastic exposure disturbs hepatic glycolipid metabolism at the physiological, biochemical, and transcriptomic levels in adult zebrafish. Sci. Total Environ..

[99] Elizalde-Velázquez G.A., Gómez-Oliván L.M. (2021). Microplastics in aquatic environments: A review on occurrence, distribution, toxic effects, and implications for human health. Sci. Total Environ..

[100] Qi Y., Yang X., Pelaez A.M., Huerta Lwanga E., Beriot N., Gertsen H., Garbeva P., Geissen V. (2018). Macro- and micro- plastics in soil-plant system: Effects of plastic mulch film residues on wheat (*Triticum aestivum*) growth. Sci. Total Environ..

[101] Vitali C., Peters R.J.B., Janssen H.-G., Nielen M.W.F. (2023). Microplastics and nanoplastics in food, water, and beverages; part I. occurrence. TrAC Trends Anal. Chem..

[102] Cverenkárová K., Valachovičová M., Mackuľak T., Žemlička L., Bírošová L. (2021). Microplastics in the Food Chain. Life.

[103] Zhang Q., Liu L., Jiang Y., Zhang Y., Fan Y., Rao W., Qian X. (2023). Microplastics in infant milk powder. Environ. Pollut..

[104] Hernandez L.M., Xu E.G., Larsson H.C.E., Tahara R., Maisuria V.B., Tufenkji N. (2019). Plastic Teabags Release Billions of Microparticles and Nanoparticles into Tea. Environ. Sci. Technol..

[105] Du F., Cai H., Zhang Q., Chen Q., Shi H. (2020). Microplastics in take-out food containers. J. Hazard. Mater..

[106] Hee Y.Y., Weston K., Suratman S. (2022). The effect of storage conditions and washing on microplastic release from food and drink containers. Food Packag. Shelf Life.

[107] Wu F., Wang Y., Leung J.Y.S., Huang W., Zeng J., Tang Y., Chen J., Shi A., Yu X., Xu X. (2020). Accumulation of microplastics in typical commercial aquatic species: A case study at a productive aquaculture site in China. Sci. Total Environ..

[108] Siddique M.A.M., Uddin A., Rahman S.M.A., Rahman M., Islam M.S., Kibria G. (2022). Microplastics in an anadromous national fish, Hilsa shad Tenualosa ilisha from the Bay of Bengal, Bangladesh. Mar. Pollut. Bull..

[109] Taghizadeh Rahmat Abadi Z., Abtahi B., Grossart H.P., Khodabandeh S. (2021). Microplastic content of Kutum fish, Rutilus frisii kutum in the southern Caspian Sea. Sci. Total Environ..

[110] Qaiser N., Sidra S., Javid A., Iqbal A., Amjad M., Azmat H., Arooj F., Farooq K., Nimra A., Ali Z. (2023). Microplastics abundance in abiotic and biotic components along aquatic food chain in two freshwater ecosystems of Pakistan. Chemosphere.

[111] Rukmangada R., Naidu B.C., Nayak B.B., Balange A., Chouksey M.K., Xavier K.A.M. (2023). Microplastic contamination in salted and sun dried fish and implications for food security—A study on the effect of location, style and constituents of dried fish on microplastics load. Mar. Pollut. Bull..

[112] Kandeyaya K.B.K.D.K., Ranatunga S., Ranatunga R.R.M.K.P. (2023). Occurrence of microplastics in some commercially important seafood varieties from Negombo, Sri Lanka. Reg. Stud. Mar. Sci..

[113] Liu Q., Chen Z., Chen Y., Yang F., Yao W., Xie Y. (2022). Microplastics contamination in eggs: Detection, occurrence and status. Food Chem..

[114] Pham D.T., Kim J., Lee S.-H., Kim J., Kim D., Hong S., Jung J., Kwon J.-H. (2023). Analysis of microplastics in various foods and assessment of aggregate human exposure via food consumption in korea. Environ. Pollut..

[115] Altunışık A. (2023). Prevalence of microplastics in commercially sold soft drinks and human risk assessment. J. Environ. Manag..

[116] Shruti V.C., Kutralam-Muniasamy G., Pérez-Guevara F., Roy P.D., Elizalde-Martínez I. (2023). First evidence of microplastic contamination in ready-to-use packaged food ice cubes. Environ. Pollut..

[117] Makhdoumi P., Amin A.A., Karimi H., Pirsaheb M., Kim H., Hossini H. (2021). Occurrence of microplastic particles in the most popular Iranian bottled mineral water brands and an assessment of human exposure. J. Water Process Eng..

[118] Zhou X.-J., Wang J., Li H.-Y., Zhang H.-M., Hua J., Zhang D.L. (2021). Microplastic pollution of bottled water in China. J. Water Process Eng..

[119] Athey S.N., Albotra S.D., Gordon C.A., Monteleone B., Seaton P., Andrady A.L., Taylor A.R., Brander S.M. (2020). Trophic transfer of microplastics in an estuarine food chain and the effects of a sorbed legacy pollutant. Limnol. Oceanogr. Lett..

[120] Chae Y., Kim D., Kim S.W., An Y.-J. (2018). Trophic transfer and individual impact of nano-sized polystyrene in a four-species freshwater food chain. Sci. Rep..

[121] Charlton-Howard H.S., Bond A.L., Rivers-Auty J., Lavers J.L. (2023). ‘Plasticosis’: Characterising macro- and microplastic-associated fibrosis in seabird tissues. J. Hazard. Mater..

[122] Pauly J.L., Stegmeier S.J., Allaart H.A., Cheney R.T., Zhang P.J., Mayer A.G., Streck R.J. (1998). Inhaled cellulosic and plastic fibers found in human lung tissue. Cancer Epidemiol. Biomark. Prev..

[123] Ragusa A., Svelato A., Santacroce C., Catalano P., Notarstefano V., Carnevali O., Papa F., Rongioletti M.C.A., Baiocco F., Draghi S. (2021). Plasticenta: First evidence of microplastics in human placenta. Environ. Int..

[124] Ragusa A., Notarstefano V., Svelato A., Belloni A., Gioacchini G., Blondeel C., Zucchelli E., De Luca C., D’Avino S., Gulotta A. (2022). Raman Microspectroscopy Detection and Characterisation of Microplastics in Human Breastmilk. Polymers.

[125] Leslie H.A., van Velzen M.J.M., Brandsma S.H., Vethaak A.D., Garcia-Vallejo J.J., Lamoree M.H. (2022). Discovery and quantification of plastic particle pollution in human blood. Environ. Int..

[126] Zhao Q., Zhu L., Weng J., Jin Z., Cao Y., Jiang H., Zhang Z. (2023). Detection and characterization of microplastics in the human testis and semen. Sci. Total Environ..

[127] Rotchell J.M., Jenner L.C., Chapman E., Bennett R.T., Bolanle I.O., Loubani M., Sadofsky L., Palmer T.M. (2023). Detection of microplastics in human saphenous vein tissue using μFTIR: A pilot study. PLoS ONE.

[128] Yang Y., Xie E., Du Z., Peng Z., Han Z., Li L., Zhao R., Qin Y., Xue M., Li F. (2023). Detection of Various Microplastics in Patients Undergoing Cardiac Surgery. Environ. Sci. Technol..

[129] Ageel H.K., Harrad S., Abdallah M.A.-E. (2022). Occurrence, human exposure, and risk of microplastics in the indoor environment. Environ. Sci. Process. Impacts.

[130] Shi X., Xu T., Cui W., Qi X., Xu S. (2023). Combined negative effects of microplastics and plasticizer DEHP: The increased release of Nets delays wound healing in mice. Sci. Total Environ..

[131] Shengchen W., Jing L., Yujie Y., Yue W., Shiwen X. (2021). Polystyrene microplastics-induced ROS overproduction disrupts the skeletal muscle regeneration by converting myoblasts into adipocytes. J. Hazard. Mater..

[132] Li Z., Zhu S., Liu Q., Wei J., Jin Y., Wang X., Zhang L. (2020). Polystyrene microplastics cause cardiac fibrosis by activating Wnt/β-catenin signaling pathway and promoting cardiomyocyte apoptosis in rats. Environ. Pollut..

[133] Lim D., Jeong J., Song K.S., Sung J.H., Oh S.M., Choi J. (2021). Inhalation toxicity of polystyrene micro(nano)plastics using modified OECD TG 412. Chemosphere.

[134] Lu L., Wan Z., Luo T., Fu Z., Jin Y. (2018). Polystyrene microplastics induce gut microbiota dysbiosis and hepatic lipid metabolism disorder in mice. Sci. Total Environ..

[135] Deng Y., Zhang Y., Lemos B., Ren H. (2017). Tissue accumulation of microplastics in mice and biomarker responses suggest widespread health risks of exposure. Sci. Rep..

[136] Jin Y., Lu L., Tu W., Luo T., Fu Z. (2019). Impacts of polystyrene microplastic on the gut barrier, microbiota and metabolism of mice. Sci. Total Environ..

[137] Li S., Shi M., Wang Y., Xiao Y., Cai D., Xiao F. (2021). Keap1-Nrf2 pathway up-regulation via hydrogen sulfide mitigates polystyrene microplastics induced-hepatotoxic effects. J. Hazard. Mater..

[138] Jiang P., Yuan G.-H., Jiang B.-R., Zhang J.-Y., Wang Y.-Q., Lv H.-J., Zhang Z., Wu J.-L., Wu Q., Li L. (2021). Effects of microplastics (MPs) and tributyltin (TBT) alone and in combination on bile acids and gut microbiota crosstalk in mice. Ecotoxicol. Environ. Saf..

[139] Wang J.J., Tian Y., Li M.H., Feng Y.Q., Kong L., Zhang F.L., Shen W. (2021). Single-cell transcriptome dissection of the toxic impact of Di (2-ethylhexyl) phthalate on primordial follicle assembly. Theranostics.

[140] Camacho L., Latendresse J.R., Muskhelishvili L., Law C.D., Delclos K.B. (2020). Effects of intravenous and oral di(2-ethylhexyl) phthalate (DEHP) and 20% Intralipid vehicle on neonatal rat testis, lung, liver, and kidney. Food Chem. Toxicol..

[141] Wang Q., Wu Y., Zhang W., Shen T., Li H., Wu J., Zhang L., Qin L., Chen R., Gu W. (2022). Lipidomics and transcriptomics insight into impacts of microplastics exposure on hepatic lipid metabolism in mice. Chemosphere.

[142] Jin H., Yan M., Pan C., Liu Z., Sha X., Jiang C., Li L., Pan M., Li D., Han X. (2022). Chronic exposure to polystyrene microplastics induced male reproductive toxicity and decreased testosterone levels via the LH-mediated LHR/cAMP/PKA/StAR pathway. Part. Fibre Toxicol..

[143] Hou B., Wang F., Liu T., Wang Z. (2021). Reproductive toxicity of polystyrene microplastics: In vivo experimental study on testicular toxicity in mice. J. Hazard. Mater..

[144] Meng X., Zhang J., Wang W., Gonzalez-Gil G., Vrouwenvelder J.S., Li Z. (2022). Effects of nano- and microplastics on kidney: Physicochemical properties, bioaccumulation, oxidative stress and immunoreaction. Chemosphere.

[145] Luo T., Zhang Y., Wang C., Wang X., Zhou J., Shen M., Zhao Y., Fu Z., Jin Y. (2019). Maternal exposure to different sizes of polystyrene microplastics during gestation causes metabolic disorders in their offspring. Environ. Pollut..

[146] Zhao T., Shen L., Ye X., Bai G., Liao C., Chen Z., Peng T., Li X., Kang X., An G. (2023). Prenatal and postnatal exposure to polystyrene microplastics induces testis developmental disorder and affects male fertility in mice. J. Hazard. Mater..

[147] Deng Y., Zhang Y., Qiao R., Bonilla M.M., Yang X., Ren H., Lemos B. (2018). Evidence that microplastics aggravate the toxicity of organophosphorus flame retardants in mice (*Mus musculus*). J. Hazard. Mater..

[148] da Costa Araújo A.P., Malafaia G. (2021). Microplastic ingestion induces behavioral disorders in mice: A preliminary study on the trophic transfer effects via tadpoles and fish. J. Hazard. Mater..

[149] Cheng W., Li X., Zhou Y., Yu H., Xie Y., Guo H., Wang H., Li Y., Feng Y., Wang Y. (2022). Polystyrene microplastics induce hepatotoxicity and disrupt lipid metabolism in the liver organoids. Sci. Total Environ..

[150] Wu B., Wu X., Liu S., Wang Z., Chen L. (2019). Size-dependent effects of polystyrene microplastics on cytotoxicity and efflux pump inhibition in human Caco-2 cells. Chemosphere.

[151] Xu M., Halimu G., Zhang Q., Song Y., Fu X., Li Y., Li Y., Zhang H. (2019). Internalization and toxicity: A preliminary study of effects of nanoplastic particles on human lung epithelial cell. Sci. Total Environ..

[152] Dong C.-D., Chen C.-W., Chen Y.-C., Chen H.-H., Lee J.-S., Lin C.-H. (2020). Polystyrene microplastic particles: In vitro pulmonary toxicity assessment. J. Hazard. Mater..

[153] Yang Y.-F., Chen C.-Y., Lu T.-H., Liao C.-M. (2019). Toxicity-based toxicokinetic/toxicodynamic assessment for bioaccumulation of polystyrene microplastics in mice. J. Hazard. Mater..

[154] Stock V., Laurisch C., Franke J., Dönmez M.H., Voss L., Böhmert L., Braeuning A., Sieg H. (2021). Uptake and cellular effects of PE, PP, PET and PVC microplastic particles. Toxicol. Vitr..

[155] Huang Z., Weng Y., Shen Q., Zhao Y., Jin Y. (2021). Microplastic: A potential threat to human and animal health by interfering with the intestinal barrier function and changing the intestinal microenvironment. Sci. Total Environ..

[156] UN Transforming Our World: The 2030 Agenda for Sustainable Development. Resolution Adopted by the General Assembly on 25 September 2015, 42809, 1–13. https://www.un.org/en/development/desa/population/migration/generalassembly/docs/globalcompact/A_RES_70_1_E.pdf.

[157] UNEP ommitting to end plastic pollution, U.S. and European Commission join Clean Seas Campaign. 2022, United Nations Environment Programme: Nairobi/Lisbon. https://www.unep.org/news-and-stories/press-release/committing-end-plastic-pollution-us-and-european-commission-join.

[158] Halfar J., Brožová K., Čabanová K., Heviánková S., Kašpárková A., Olšovská E. (2021). Disparities in Methods Used to Determine Microplastics in the Aquatic Environment: A Review of Legislation, Sampling Process and Instrumental Analysis. Int. J. Environ. Res. Public Health.

[159] Kish R.J. (2018). Using legislation to reduce one-time plastic bag usage. Econ. Aff..

[160] European Union Commission Plastics Strategy. https://environment.ec.europa.eu/strategy/plastics-strategy_en.

[161] European Union Commission (2022). Circular Economy: Commission Takes Action to Reduce Waste from Single-Use Plastics.

[162] Union E. Commission Delegated Decision (EU) 2024/1441 of 11 March 2024 supplementing Directive (EU) 2020/2184 of the Euripean Parliament and of the Council by laying down a methodology to measure microplastics in water intended for human consumption. https://eur-lex.europa.eu/eli/dec_del/2024/1441/oj.

[163] Zhou Y., Ashokkumar V., Amobonye A., Bhattacharjee G., Sirohi R., Singh V., Flora G., Kumar V., Pillai S., Zhang Z. (2023). Current research trends on cosmetic microplastic pollution and its impacts on the ecosystem: A review. Environ. Pollut..

[164] Mamun A.A., Prasetya T.A.E., Dewi I.R., Ahmad M. (2023). Microplastics in human food chains: Food becoming a threat to health safety. Sci. Total Environ..

